# Topological Filtering of Dynamic Functional Brain Networks Unfolds Informative Chronnectomics: A Novel Data-Driven Thresholding Scheme Based on Orthogonal Minimal Spanning Trees (OMSTs)

**DOI:** 10.3389/fninf.2017.00028

**Published:** 2017-04-26

**Authors:** Stavros I. Dimitriadis, Christos Salis, Ioannis Tarnanas, David E. Linden

**Affiliations:** ^1^Institute of Psychological Medicine and Clinical Neurosciences, School of Medicine, Cardiff UniversityCardiff, UK; ^2^Cardiff University Brain Research Imaging Center (CUBRIC), School of Psychology, Cardiff UniversityCardiff, UK; ^3^School of Psychology, Cardiff UniversityCardiff, UK; ^4^Neuroinformatics.GRoup, School of Psychology, Cardiff UniversityCardiff, UK; ^5^Department of Informatics and Telecommunications Engineering, University of Western MacedoniaKozani, Greece; ^6^Health-IS Lab, Chair of Information Management, ETH ZurichZurich, Switzerland; ^7^3rd Department of Neurology, Medical School, Aristotle University of ThessalonikiThessaloniki, Greece; ^8^Neuroscience and Mental Health Research Institute (NMHRI), School of Medicine, Cardiff UniversityCardiff, UK

**Keywords:** EEG, fMRI, resting state, graph theory, phase locking value, dynamic functional connectivity, minimal spanning tree, topological filtering

## Abstract

The human brain is a large-scale system of functionally connected brain regions. This system can be modeled as a network, or graph, by dividing the brain into a set of regions, or “nodes,” and quantifying the strength of the connections between nodes, or “edges,” as the temporal correlation in their patterns of activity. Network analysis, a part of graph theory, provides a set of summary statistics that can be used to describe complex brain networks in a meaningful way. The large-scale organization of the brain has features of complex networks that can be quantified using network measures from graph theory. The adaptation of both bivariate (mutual information) and multivariate (Granger causality) connectivity estimators to quantify the synchronization between multichannel recordings yields a fully connected, weighted, (a)symmetric functional connectivity graph (FCG), representing the associations among all brain areas. The aforementioned procedure leads to an extremely dense network of tens up to a few hundreds of weights. Therefore, this FCG must be filtered out so that the “true” connectivity pattern can emerge. Here, we compared a large number of well-known topological thresholding techniques with the novel proposed data-driven scheme based on orthogonal minimal spanning trees (OMSTs). OMSTs filter brain connectivity networks based on the optimization between the global efficiency of the network and the cost preserving its wiring. We demonstrated the proposed method in a large EEG database (*N* = 101 subjects) with eyes-open (EO) and eyes-closed (EC) tasks by adopting a time-varying approach with the main goal to extract features that can totally distinguish each subject from the rest of the set. Additionally, the reliability of the proposed scheme was estimated in a second case study of fMRI resting-state activity with multiple scans. Our results demonstrated clearly that the proposed thresholding scheme outperformed a large list of thresholding schemes based on the recognition accuracy of each subject compared to the rest of the cohort (EEG). Additionally, the reliability of the network metrics based on the fMRI static networks was improved based on the proposed topological filtering scheme. Overall, the proposed algorithm could be used across neuroimaging and multimodal studies as a common computationally efficient standardized tool for a great number of neuroscientists and physicists working on numerous of projects.

## Introduction

The brain is an inherently dynamic system which both resting-state networks (Hansen et al., 2015) and networks connected to executive cognition requires dynamic reconfiguration of highly evolving networks of brain areas that interact via transient complexity patterns (Bassett et al., 2011; Braun et al., 2015a). To investigate how the brain functionality changes over experimental time, *time-varying graphs* should be computed as networks that evolve over time with fixed number of nodes but links that change over time. The dynamic approach of brain connectivity is explored by adopting a time-window over experimental time and constructs quasi-static connectivity graphs from the temporal signal segments enclosed within this time-window where various network metrics can be estimated for each window leading to network metric time series (NMTS) (Dimitriadis et al., 2010a, 2012a, 2013a,b, 2015a,c; Sporns, 2011, 2014).

Both static and dynamic graphs can be analyzed either as binary or weighted networks after first applying a thresholding criterion. With both full binary and weighted graphs, every pair of brain areas is directly connected. Many researchers have argued that full-weighted graphs are not “true” networks since brain areas are inter-connected via sparse anatomical connections (Sporns, 2011). A recent study supported that weighted graphs are also less computationally efficient, when someone attempts to analyze large-scale networks like the well-known voxel-based functional connectivity networks (Telesford et al., 2011). Also the overrepresentation of functional connections between brain areas added difficulties to the extraction of significant topological information (Serrano et al., 2009).

To work either with binary or weighted graphs, a large number of topological thresholding schemes can be applied. The list of available thresholding schemes can be divided into arbitrary and data-driven methods. Arbitrary thresholding schemes includes (1) the preservation of the same degree (number of connections per node), *k*, for each graph, so that the subsequently derived network metrics are comparable across graphs/subjects/conditions (Milo et al., 2002; Sporns and Zwi, 2004; Stam et al., 2007a; Dimitriadis et al., 2009; Micheloyannis et al., 2009), (2) the maintenance of a specific ratio of the strongest edges (sparsity) (Stam et al., 2007a; Rubinov et al., 2009) and (3) the absolute threshold up to a predefined value (between 0 and 1). Each approach has both advantages and disadvantages. The aforementioned thresholding schemes even though are simplified, adds bias for group and task comparisons and moreover, reduce the possibility of the reproducibility of the findings across studies from different research groups but also within the same research group with the extension of recording sessions. Absolute thresholds set a value between 0 and 1 above which the strength of connections survived while below are excluded from further analysis and are set to 0. Proportional thresholds employ a % percentage of the strongest connections (edges), like the top 10% of weighted values in the network. The preservation of the same degree, *k*, for each graph is more meaningful compared to the two aforementioned approaches since it picks equiprobable strong and weak weights but also assumed that networks have the same mean degree across group and tasks.

Even though connectedness in a node level between groups and conditions could be informative, it restricts both the interpretation and the comparison between various graph measures that vary with the degree (Alexander-Bloch et al., 2013). Additionally, absolute thresholding scheme divides the weights of connections into two groups, weak and strong connections emphasizing either the weak or the strong (van Wijk et al., 2010). A number of studies attempted to diminish the effect and the reproducibility of their results due to the adopted thresholding scheme by presenting various network measures over traditional cumulative thresholding. Specifically, for absolute, sparsity (proportional) and mean degree thresholding schemes, they demonstrated their network-oriented results over a range of values (0–1 for absolute, 2–40% for sparsity, 1-(N-1) for degree preservation where N denotes the number of nodes). Based on correlation coefficient r, a range of thresholds has been applied, e.g., *r* = 0.1 (Buckner et al., 2009) and *r* = 0.8 (Tomasi and Volkow, 2010). For sparsity, a range of proportional thresholds have been presented, e.g., from 5 to 40% (e.g., Fornito et al., 2011) or for a narrower range of values in Alzheimer's disease (Stam et al., 2009) and in schizophrenia (Micheloyannis et al., 2006). One of the very first studies that presented network metrics over a range of degree was a MEG resting-state study at both control and Alzheimer's disease group (Stam et al., 2007a) and also in EEG sleep study (Dimitriadis et al., 2009). A few researchers attempted to present their results in narrower range of thresholds in order to support the insensitivity of their results to the arbitrary choice of the threshold (e.g., Cole et al., 2013, 2014, top 2–10%; van den Heuvel et al., 2009, *r* = 0.3–0.5). However, the above approach will lead to unstable results if the adopted network properties are sensitive to a large range of thresholds, especially in the case where group or task differences in terms of network metrics are reversed within a range of a threshold (e.g., Scheinost et al., 2015). An alternative solution to traditional cumulative thresholding scheme is the alternative windowed thresholding scheme (Bassett et al., 2012) where the connections were binning into 100 bins and finally 100 graphs were created where the first graph encapsulated the 1% of strongest connections, the second graph the 1% of the next strongest connections etc. (Bassett et al., 2012). Finally, a few studies fairly demonstrated their results, e.g., hub detection that is consistent over a range of absolute thresholds (Buckner et al., 2009).

According to our knowledge, two data-driven thresholding schemes exist in the literature. The first one has as an objective criterion to maximize the formula of cost-efficiency (cost efficiency = global efficiency − cost) of a network vs. its cost (the ratio of the existing edges divided by the total number of possible edges) that typically range between the upper and lower limits of an economical small-world network (Bassett et al., 2008). The basic drawback of this method is that it accumulates edges based on their strength and applying an iteratively absolute threshold searching approach from 0 to 1 without taking into consideration any topological constraint. Additionally, the aforementioned approach completely separate weak from strong connections where in some diseases studying the mixture of both strong and weak connections can improve the designing of reliable connectomic biomarkers (Bassett et al., 2012). The second data-driven thresholding scheme based on the notion of shortest path lengths (SPL) and assumes that the information between brain areas flows via the shortest pathways (Dimitriadis et al., 2010a).

Minimal spanning tree (MST) is a unique representation of a functional brain network and it is a unique acyclic subgraph that connects all nodes and maximizes the synchronization between brain areas. MST has already been applied in human brain networks where both trivial network metrics but also tree-oriented properties have been estimated based on the unique sparse MST-representation of a weighted graph (Vourkas et al., 2014; for a review see Stam, 2014; Stam et al., 2014).

Current approach attempted to maximize the information flow over the network vs. the cost by selecting the connections via the notion of orthogonal minimal spanning trees (OMSTs). OMSTs based on the notion of sampling the full-weighted brain network over consecutive rounds of MST that are orthogonal to each other. Practically, we extracted the 1st MST, then we zeroing their connections and we estimate the 2nd MST based on the rest of the network. With this iteratively approach, we can get orthogonal MST and topologically filtering brain network by optimizing the global efficiency of the network constrained by the cost of keeping its connections.

Therefore, this study sought to introduce a novel data-driven thresholding scheme based on OMSTs that will advance the ability of research neuroimaging centers to compare and analyse large imaging connectomes/connectomics under a common topological filtering framework. This will enforce our ability to compare results from different studies based on the same imaging method and condition and also between different imaging methods in a multimodal imaging approach.

Test-retest reliability of the network metrics derived from functional networks is of significant value in the neuroscience community (Zuo et al., 2012, 2014; Zuo and Xing, 2014; Chen et al., 2015). Many researchers world-wide presented connectomic biomarkers for various brain disorders/diseases and for that reason, their reproducibility should be explored (Kaiser, 2013; Dimitriadis et al., 2015b,c,d, 2016a,b). The choice of the appropriate preprocessing steps to achieve reproducible measurements is more than significant. One significant preprocessing step in the choice of topological filtering scheme to untangle the “true” backbone of a brain network. Therefore, we validated the proposed data-driven topological filtering OMSTs method and the rest in terms of intra-class correlation (ICC) of basic network metrics derived from static functional networks estimated from resting-state BOLD activity. For that reason, we employed an open fMRI database from a single-subject that has been scanned 100 times.

To validate its potentiality, a large EEG study of resting-state activity was used in order to increase the recognition accuracy of each subject over the rest of the database based on nodal network metric time series (nNMTS) using well-known network metrics (Dimitriadis et al., 2010a). The EEG database is a free available dataset repository as part of PhysioNet BCI Database^1^. The whole analysis based on dynamic functional brain connectivity and a set of nNMTS over frequencies and topology (EEG sensors) which were used as the unique brain fingerprinting of each subject (Finn et al., 2015). Our method was compared to various thresholding schemes based on the recognition accuracy of each individual based on the nNMTS signature to the rest of cohort. Additionally, since the EEG dataset based on a single-trial, we accessed the reliability of the current method and the derived network metrics from a free available dataset of a case study of fMRI resting-state recordings repeated over 100 scans (Poldrack et al., 2015). The fMRI dataset from the single-case long term study can be downloaded and preprocessed with available code from the author's website^2^.

## Materials and methods

On this section, we described both free available neuroimaging datasets (EEG and fMRI) with the adopted preprocessing steps. The main goal with EEG resting-state dataset was to demonstrate the superiority of the proposed topological filtering scheme to filter dynamic networks using as validation criterion the recognition accuracy of each subject to the rest of the cohort. Using multiple scans of fMRI, we demonstrated the superiority of the topological filtering method to increase the reliability of trivial network metrics across scans.

### EEG recordings

Scalp EEG signals were gathered from the freely online database PhysioNet BCI (Database physionet BCI^1^ Schalk et al., 2004). The database consists of *N* = 101 healthy subjects recorded in two different baseline conditions, i.e., 1-min eyes-open (EO) resting state and 1-min eyes-closed (EC) resting state. In each condition, subjects were comfortably seated on a reclining chair in a dimly lit room. During EO they were asked to avoid ocular blinks in order to reduce signal contamination. The EEG data were recorded with a 64-channel system (BCI2000 system (BCI2000 system) with an original sampling rate of 160 Hz.

### Preprocessing

Ongoing activity was corrected for artifacts through a two-step procedure implemented in Matlab (The MathWorks, Inc., Natick, MA, USA) and Fieldtrip (Oostenveld et al., 2011). Line noise was first removed using a notch filter at 60 Hz and the single-subject data was whitened and reduced in dimensionality by means of Principal Component Analysis (PCA) with a threshold corresponding to 95% of total variance (Delorme and Makeig, 2004; Escudero et al., 2011; Antonakakis et al., 2013). The resulting signals were submitted to independent component analysis (ICA) using the extended Infomax algorithm as implemented in EEGLAB (Delorme and Makeig, 2004). A given independent component was considered to reflect ocular or cardiac artifacts if more than 30% of its z-score kurtosis or skewness values, respectively, were outside ±2 of the distribution mean (Escudero et al., 2011; Antonakakis et al., 2013; Dimitriadis et al., 2014). The remaining ICs were used to reconstruct a relatively artifact-free signal. The average number of artifactual ICs was 5.5 for eyes-closed and 5.1 for the eyes-open condition.

### Functional connectivity

Here, functional connectivity was examined among the following 8 brain rhythms where the optimal low frequency for phase *f*φ was in one of the typical sub-bands of electrophysiological neural signals {δ, θ, α_1_, α_2_, β_1_, β_2_, γ1, γ2}, defined respectively within the ranges {0.5–4 Hz; 4–8 Hz; 8–10 Hz; 10–13 Hz; 13–20 Hz; 20–30 Hz; 30–48 Hz; 52–70 Hz}. We adopted a 3rd order Butterworth filters applied in a zero-phase mode to get the characteristic brain rhythms. Among the available connectivity estimators, we adopted the one based on the imaginary part of phase-locking value (iPLV) (Lachaux et al., 1999) and adjusted properly so as to extract time-resolved profiles of intra-frequency coupling from EEG multichannel recordings at resting state.

The original PLV is defined as follows:

(1) $$\begin{array}{l} PLV=\frac{1}{T}*\overset{T}{\underset{t=1}{\sum }}e^{i\left(\phi_{k}\left(t\right)-\phi_{l}\left(t\right)\right)} \end{array}$$

Where *t* refers to time in samples while φ to the phase time series extracted via the Hilbert Transform and {*k, l*} to the EEG sensors.

while the imaginary part of PLV as follows:

(2) $$\begin{array}{l} ImPLV=\frac{1}{T}*|Im\left(\overset{T}{\underset{t=1}{\sum }}e^{i\left(\phi_{k}\left(t\right)-\phi_{l}\left(t\right)\right)}\right)| \end{array}$$

The imaginary part of PLV (iPLV) investigates intra-frequency interactions without putative contributions from volume conductance. In general, the iPLV is mainly sensitive to non-zero-phase lags and for that reason is resistant to instantaneous self-interactions from volume conductance (Nolte et al., 2004). In contrast, it could be sensitive to phase changes that not necessarily imply a PLV oriented coupling.

#### Dynamic iPLV estimates: the time-varying iPLV graph (^TV^iPLV graph)

The goal of the analytic procedures described in this section was to understand the repertoire of phase-to-phase interactions and their temporal evolution, while taking into account the quasi-instantaneous spatiotemporal distribution of iPLV estimates. This was achieved by computing one set of iPLV estimates within each of a series of sliding overlapping windows spanning the entire 1-min continuous EEG recording for both eyes-open and closed condition. The width of the temporal window was set equal to the duration of ten cycles of each frequency band. The center of the stepping window moved forward every 20 ms and the intra-frequency interactions between every possible pair of frequencies were reestimated leading to a quasi-stable in time static iPLV graph. In this manner, a series of 1,496 for δ to 1,930 for γ_2_ sets of iPLV graph estimates were computed per condition, frequencies and for each participant.

This procedure, the implementation details of which can be found elsewhere (Dimitriadis et al., 2010b, 2012a, 2013a, b, 2015a, c), resulted in 8 time-varying iPLV graphs per participant (^TV^iPLV), each serving as an instantaneous snapshot of the surface network. ^TV^iPLV tabulates iPLV estimates between every possible pair of sensors. For each subject, a 4D tensor (frequencies bands x slides x sensors x sensors) was created for each condition integrating subject-specific spatio-temporal phase interactions.

#### Surrogate data analysis of iPLV estimates—statistical filtering of brain networks

To identify significant iPLV-interactions which were estimated for every pair of frequencies within and between all 64 sensors, and at each successive sliding window (i.e., temporal segment), we employed a surrogate data analysis (Theiler et al., 1992). Accordingly, we could determine (a) if a given iPLV value differed from what would be expected by chance alone, and (b) if a non-zero iPLV corresponded to non-spurious coupling.

For every temporal segment, sensor-pair, and frequency, we tested the null hypothesis H_0_: “the observed iPLV value comes from the same distribution as the distribution of surrogate iPLV-values.” One thousand surrogate time-series were generated by cutting them at a single point at a random location and exchanging the two resulting time courses (Aru et al., 2014). We restricted the range of the selected cutting point in a temporal window of width equals to 10 s in the middle of the recording session (between 25 and 35 s). This surrogate scheme was applied to the original whole time series and not to the signal-segment at every slide. Repeating this procedure leads to a set of surrogates with a minimal distortion of the original phase dynamics while destroying less the non-stationarity of the brain activity compared to shuffling the time series or cutting and rebuilding it in more than one time points. Using the method of delay vector variance (DVV), we estimated the non-linearity/non-stationarity of the surrogate time series which didn't differ statistically with the original time series (see S1 in Supplementary Material; Gautama et al., 2004).

This procedure assures that the real and surrogate indices both have the same statistical properties. For each data set the surrogate iPLV (^s^iPLV) was then computed. We then determined a one-sided *p*-value for each iPLV value that corresponded to the likelihood that the observed value could belong to the surrogate distribution. This was done by directly estimating the proportion of “surrogate” iPLV^s^ that were higher than the observed iPLV (Theiler et al., 1992). The *p*-value reflected the statistical significance of the observed iPLV-level (a very low value revealed that it could not have appeared from processes with no iPLV coupling).

The FDR method (Benjamini and Hochberg, 1995) was employed to control for multiple comparisons (across all possible pairs of frequencies) with the expected fraction of false positives set to *q* ≤ 0.01. Finally, for each subject the resulting ^TV^iPLV profiles constituted a 4D array of size [8 (number of frequencies) × 1,896 (time windows) × 64 (sensors) × 64 (sensors)] with a value of 0 indicated a non-significant iPLV value.

#### Network metric time series (NMTS): dynamic topological properties of the underlying brain networks

Many well-known topological metrics can be estimated over dynamic networks (Rubinov and Sporns, 2010). Here, we used global efficiency (GE) for weighted networks of *NxN* nodes which expressed the inverse of the shortest path length between every possible pair of nodes and provides an index of the information transfer in the network (Latora and Marchiori, 2001; Achard and Bullmore, 2007). GE is defined as follows:

(3) $$GE=\frac{1}{N}\sum_{i\in N}\frac{\sum_{j\;\in \;N,\;j\;\neq \;i}{\left(d_{ij}\right)}^{-1}}{N-1}$$

with *N* representing the number of nodes (sensors or ROIs) in the network, *w*_*ij*_ the weights between nodes and *E* the total number of edges.

### Graph construction

In order to perform any type of multivariate brain network analysis described aforementioned, it is significant to threshold the full-weighted correlation matrices to more sparse and meaningful binary or weighted graphs by applying one or a set of thresholding schemes (Bullmore and Bassett, 2011). A set of brain networks from a target group can be threshold to extract equi-sparse graphs by applying to each subject different thresholds in order to ensure that the brain networks of all the subjects have the same sparsity, the same number of edges (Achard et al., 2006; Bassett and Bullmore, 2006) or equi-threshold graphs where the brain network of each individual has a different number of connections (van den Heuvel et al., 2008; van den Heuvel et al., 2009; Hayasaka and Laurienti, 2010). In both the aforementioned cases, a network topology is examined over a large range of sparsity values, e.g., between 0.1 (keeping 10% of connections) to 0.5 with a stepping criterion of 0.01 (Bassett and Bullmore, 2009; Bullmore and Sporns, 2009; Bullmore and Bassett, 2011).

#### An overview of topological filtering of brain networks

**Figure 1:**
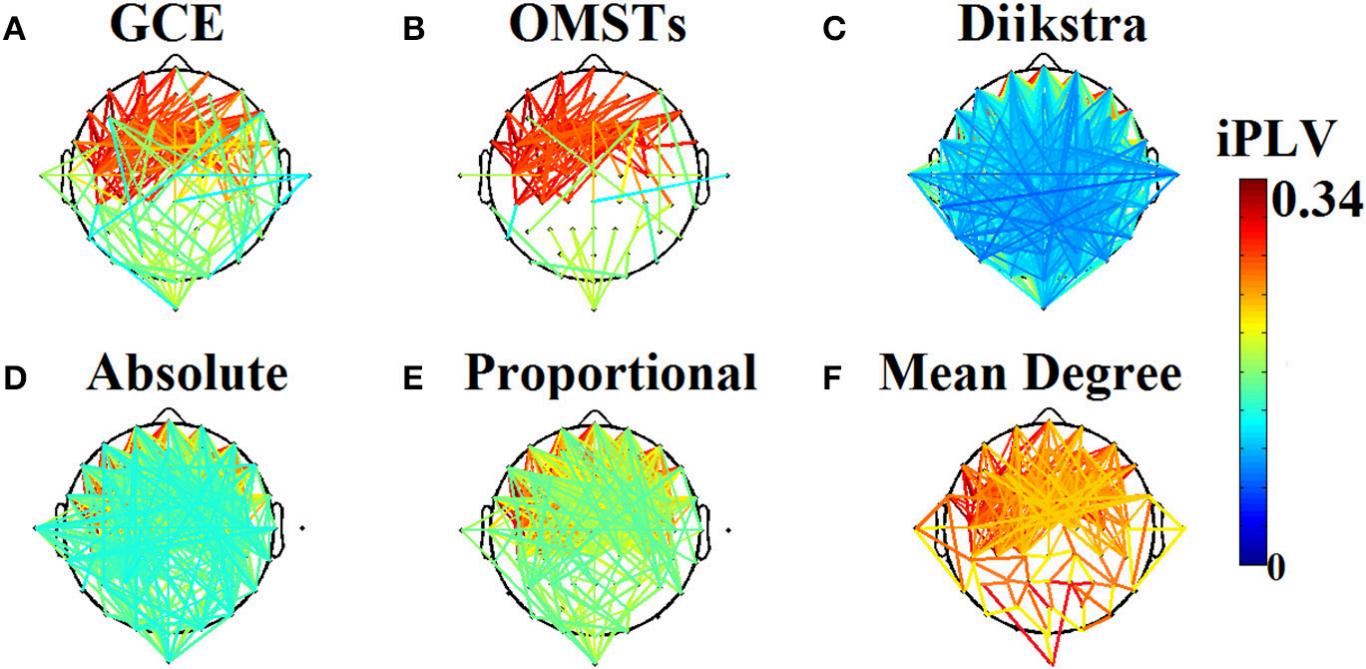
**Topological layouts of the six thresholding schemes applied to a static functional connectivity graph (FCG) from δ frequency bands during eyes-open condition**. **(A)** Global Cost Efficiency (GCE), **(B)** Orthogonal Minimal Spanning Trees (OMSTs), **(C)** Dijkstra's algorithm, **(D)** Absolute threshold, **(E)** Proportional Thershold, **(F)** Mean Degree Threshold. Panels **(A–C)** are data-driven topological filtering schemes while **(D–F)** are arbitrary thresholding schemes.

The majority of neuroimaging studies that explored functional brain connectivity in various tasks, conditions and in both healthy and disease groups employed three basic thresholding schemes. The most common thresholding schemes are the following:

1. Mean degree (Milo et al., 2002; Sporns and Zwi, 2004; Stam et al., 2007a; Dimitriadis et al., 2009; Micheloyannis et al., 2009),
2. The sparsity, the maintenance of a specific ratio of the strongest edges (Stam et al., 2007a,b; Rubinov et al., 2009),
3. The selection of a % of the strongest connections over the whole graph,
4. The maximization of the following formula cost efficiency = global efficiency − cost) (Bassett et al., 2008), and
5. A data-driven algorithm based on Dijkstra algorithm (Dimitriadis et al., 2010b). Figure 1 demonstrates the topology of the connections survived after applying the six different thresholding schemes (including the proposed based on OMST) over a weighted graph from a single subject in δ band in eyes-open condition. It is clear that the top-3 data-driven thresholding schemes select connections with a larger range of values and a mixture of both strong and lower connection values (Figures 1A–C) while the bottom-3 arbitrary thresholding schemes extract mostly the strongest connections (Figures 1D–F).

Apart from analyzing topological network metrics over a range of absolute, mean degree and sparsity using cumulative thresholding procedure, there is a new approach based on windowed thresholding. To overcome this weakness, a recent study proposed to retain connections that fell within a range of strength rather than above a threshold (Schwarz and McGonigle, 2011). With windowed thresholding one can examine independent sets of connections while the traditional cumulative thresholding allows for the examination of non-independent sets of connections.

#### Identification of significant edges based on Dijkstra's algorithm

In a previous study, we introduced a novel, unbiased technique to access the most significant edges of a weighted network based on Dijkstra's algorithm (Dijkstra, 1959). This algorithm constituted a heuristical approach that alleviated the need for defining a threshold according to any particular optimization scheme. The application of Dijkstra's algorithm to the point-wise inverse of the connectivity matrix corresponding to an FCG resulted in the path of lowest traveling cost between every possible pair of nodes. Two different matrices were derived with this procedure: (1) a weighted graph denoted as a *distance matrix*, with all the shortest path lengths' inter-nodes that tabulated the traveling costs, and (2) an *adjacency matrix* that kept the weights of all the edges utilized in the formation of the shortest paths (see Figure 1C).

### fMRI single-case long term dataset

#### Description of the scanning protocol

The participant on this single-case study (author R.A.P.) is a right-handed Caucasian male, aged 45 years at the onset of the study. He suffers from plaque psoriasis but is otherwise generally healthy. RS-fMRI was performed in 100 scans throughout the data collection period (89 in the production phase), using a multi-band EPI sequence (TR = 1.16 ms, TE = 30 ms, flip angle = 63 degrees (the Ernst angle for gray matter), voxel size = 2.4 × 2.4 × 2 mm, distance factor = 20%, 68 slices, oriented 30 degrees back from AC/PC, 96 × 96 matrix, 230 mm FOV, MB factor = 4, 10:00 scan length). Freesurfer parcellation of BOLD activity gave a total of 630 regions for subsequent analysis. For further details please see the original paper (Poldrack et al., 2015).

#### Graph construction

The maximal overlap discrete wavelet transform (MODWT) method has been used to create functional connectivity matrices (Deuker et al., 2009). Our analysis based on 0.06~0.125 Hz (Scale 2). Bold activity of each of the 630 regions was decomposed with MODWT in wavelet coefficients. Using correlation coefficient in a pair-wise fashion between the wavelet coefficients of every pair of the regions, we estimated a single static functional connectivity graph (FCG) with dimensions 630 × 630 for each scan. The whole analysis was repeated for all the trials. Finally, we got the absolute values of the correlation-based FCGs and we normalized with the maximum observed value to range the FCG into [0,1].

#### Network analysis and reproducibility

Two basic network metrics were estimated from the single-trial FCGs, the global and local efficiency. The ICC was estimated as a measure of test–retest reliability for both graph metrics under multiple trials (scans). A value close to 1 means that the estimated network metrics are consistent across scans. We optimized the three arbitrary thresholding schemes (mean degree, absolute threshold and % of strongest connections (proportional) over the maximization of the sum of ICC for global and local efficiency.

## A data-driven thresholding scheme based on orthogonal minimal spanning trees (OMSTs)

### Minimal spanning tree (MST) algorithm

In graph theory, a tree is defined as an acyclic connected graph (Estrada, 2011). Acyclic means that there are no loops (of any length) in the graph. A graph is connected if there exists a path between each pair of nodes in the graph. A tree with *N* nodes has exactly *m* = *N* − 1 links or edges. A spanning tree is a subgraph that includes all nodes of the original graph (it has the same *N*) but only *N* − 1 edges (it has no cycles). A minimum spanning tree (MST) of a connected weighted graph is the spanning tree of this graph that minimizes the sum of the weights of the edges included in the tree. If all the weights in the weighted graph are unique, its MST is also unique (Mares, 2008). In other words, there is only one MST that corresponds to a weighted graph with unique weights.

Two major algorithms have been described to construct the MST of a weighted graph (Kruskal, 1956; Prim, 1957). Here, we used Kruskal's algorithm. Prim's method produces the same MST if the weights of the original graph are unique. The running time of the MST is O((V + E)Â ·logV) where E denotes edges and N the vertices.

### Orthogonal minimal spanning tree (OMSTs)

It was proved that MST is an unbiased method for brain networks in order to get reliable network metrics (Tewarie et al., 2014). In contrast, MST for large brain networks of hundreds of nodes is a very sparse network that cannot always capture the true topology and can diminish the strength of discriminating two groups. Recent studies demonstrated the efficacy of brain networks and machine learning techniques for discriminating a control and one (e.g., mTBI; Dimitriadis et al., 2015c; Antonakakis et al., 2016) or more target groups (e.g., mild cognitive impairment and Alzheimer's disease; Supekar et al., 2008; Brier et al., 2013; Khazaeea et al., 2017). All the aforementioned studies adopted a data-driven topological filtering method before extracted network metrics as features for the classification. Another study used MST while adding connections from the rest of the network using as criterion the proportion of functional connections into four sparsity levels in order to demonstrate the effect on the results (Song et al., 2015).

Usage of the orthogonal MST graph leads to a better sampling of brain network preserving the advantage of MST that connects the whole network with minimum cost without introducing cycles and without differentiated strong from weak connections.

**Figure 2:**
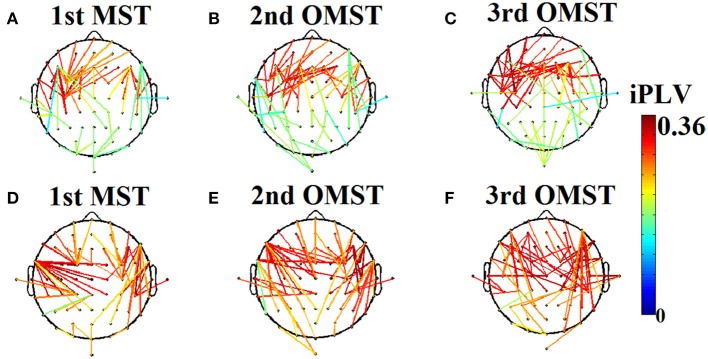
**An application of Orthogonal Minimal Spanning Tree (OMSTs) applied to a static functional connectivity graph (FCG) from δ frequency bands during eyes-open condition (A–C)** and eyes-closed condition **(D–F)**.

OMST is estimated as follows: The initial 1st MST connects all the N nodes by using *N* − 1 edges. Then, the *N* − 1 connections of the 1st MST will substituted with zeros and a 2nd -MST is estimated that connects all of the N points with minimal total distance, satisfying the constraint that is orthogonal—i.e., shares no common edges- to the 1st MST. Afterward, the *N* − 1 connections of the 2nd MST will substituted with zeros and a 3rd −MST will be estimated that connects the nodes with the minimal total weight, subject to the constraint that it is orthogonal to the previous two constructed (1st and 2nd) MST's. In general, an m-MST is orthogonal to all the previous (m − 1) MST's, having exactly m·(*N* − 1) edges (Figure 2). The whole computational times is equal to O(m^*^(N + E)Â ·logV).

Figure 2 demonstrates the MST (Figures 2A,D) and the first two OMST (Figures 2B,C), and (Figures 2E,F) applied to a static graph from a single subject at δ band from eyes-open (Figure 2A–C) and eyes-closed condition (Figures 2D–F).

### The proposed algorithm based on OMSTs

It is important to mention here that OMST should be worked on the inverse weighted graph (distance matrix) so as to capture the strongest connections which can be interpreted that two brain areas are more functionally “closed.”

Our data-driven thresholding algorithm based on OMSTs works as followed:

- We extract the OMSTs by applying iteratively the Kruskal's algorithm on the inversed functional brain graph because we want to collect the most significant connections under the constraint of MST.
- After extracting the 1st MST, we substituted the *N* − 1 edges with ‘Inf’ in the original network in order to avoid capturing the same edges and also to keep the orthogonality of the next MST.
- We aggregated connections over the OMSTs (including the 1st) so as to optimize the formula global efficiency − cost vs. cost. This procedure can employ, e.g., 3^*^(*N* − 1) edges from the first three OMSTs plus 10 edges from the 4th OMSTs.
- For each adding connection, we estimated the objective function of Global Cost Efficiency (GCE) = global efficiency − cost) where cost denotes the ratio of the total weight of the existing edges divided by the total strength of the original full-weighted graph. The values of this formula range within the limits of an economical small-world network for healthy control participants (Bassett and Bullmore, 2006).

Our criterion to topologically filter a given brain network is by finding the maximum value of the following quality formula:

(4) $$\begin{array}{l} J_{GCE}^{OMSTs}=GE-Cost \end{array}$$

A recent study based on original and artificial biological networks demonstrate that wiring cost supports the evolution of both modular and hierarchical organization of biological networks (Mengistu et al., 2016). Additionally, these biological networks exhibit higher performance in terms of information flow and adoptability to new environments. Complementary to previous studies that demonstrate the relationship between sparsity and hierarchy (Corominas-Murtra et al., 2013), this study explained and validated why sparsity leads to hierarchy under the force of wiring cost and provides new information about the evolution of hierarchy (Mengistu et al., 2016). For that reason, both global efficiency as an index of how efficient the network operates and the cost should be part of the optimized function J.

**Figure 3:**
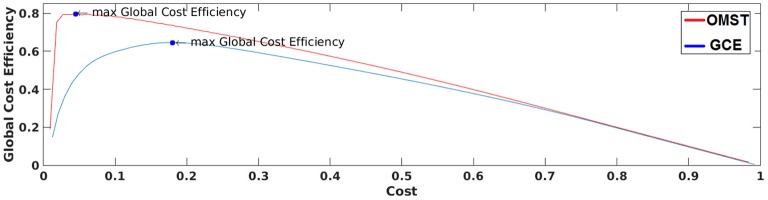
**A demonstration of how GCE algorithm (GCE) and the proposed Orthogonal Minimal Spanning Tree (OMST) data-driven thresholding scheme are working applied to a static functional connectivity graph (FCG) from δ frequency bands during eyes-open condition**. Blue circles denote the maximization of global cost efficiency with the two approaches.

Figure 3 demonstrates how our algorithm works in comparison with algorithm proposed in Bassett and Bullmore (2006) from a single subject at δ band from the eyes-open condition. The basic difference of these two algorithms is the sampling of connections from the given brain network. Our approach based on OMST while the one used by Bassett et al., on iteratively absolute thresholding the weights of functional connections without any topological criterion and distinguishes weak from strong connections. The two curves represented the quality formula in Equation (4) over various costs using the two sampling approaches. Finally, we extracted as optimal thresholding functional brain network the one that maximizes the formula in Equation (4) (see the arrow in Figure 3). We can clearly see in Figure 3 that OMST optimizes better the topological criterion compared to Bassett's approach.

## Unique brain fingerprinting based on network metric time series (NMTS)

For each of the aforementioned thresholding schemes (absolute, proportional, mean degree, shortest-path edges selection and the maximization of global cost efficiency) and the proposed one based on OMSTs, we estimated nodal global efficiency across time based on the thresholding versions of dynamic 4D ^TV^iPLV arrays across subjects and frequency bands. This approach results to subject and frequency specific nNMTS^GE^ from GE which will then be used as possible brain fingerprinting features.

To access the recognition accuracy of each node and network metric over the eight frequency bands, we split each nNMTS^GE^ into two equal segments and we employed **Wald-Wolfowitz (WW) test** as a similarity index to each target nNMTS^GE^ from the database. On the next section, we described how WW test was used.

### A dissimilarity measure for dynamical trajectories based on the wald-wolfowitz (WW) test

The two-sample, non-parametric WW test was adopted in the present work to assess the degree of similarity between two nodal network metric time series based on global efficiency (nNMTS^GE^) derived from dynamic FCGs. The procedure entailed, first, transforming every pair of NMTS^GE^ time series *x(t), t* = 1.2,…T into dynamic trajectories represented by multidimensional vectors *X*_*t*_ = [*x*(*t*), *x*(*t* + 1),…, *x*(*t* + *d*_*e*_)] and *Y*_*t*_ = [*y*(*t*), *y*(*t* + 1),…, *y*(*t* + *d*_*e*_)] (*X* and *Y* correspond to two split-half segments from a single participant or from two participants). These vectors were formed by selecting the appropriate set of *d*_*e*_, which is the embedding dimension parameter that controls the dimensionality of the vectors and *d*_*t*_ is the time-delay. By adopting the Ragwitz criterion, we optimized the embedding dimension *d*_*e*_ and the embedding delay *d*_*t*_ (Ragwitz and Kantz, 2002), resulting in values ranging from 3 to 6 in both the complete and split-half temporal segments of NMTS^GE^ series. The two point-samples {X_*t*_}_*t* = 1:*m*_ and {Y_*t*_}_*t* = 1:*n*_ were then formed and the w_dist_ = w({X_*t*_},{Y_*t*_}) was computed.

Next, the minimal spanning tree (MST) graph of the overall sample was constructed (i.e., disregarding the sample identity of each point). In these graph points represent nodes with N − 1 edges (N = n + m) (i.e., paths within each pair of nodes). The second step of the procedure entails computing the R statistic which is the total number of consecutive sequences with identical sample identities (i.e., “runs”). Based on the number of edge pairs of MST sharing a common node and the degrees of the nodes, the mean and variance of R can be calculated (Laskaris and Ioannides, 2001). This property of R permits computation of the initial form of the normally-distributed, WW Dissimilarity Index (w) as follows:

(5) $$\begin{array}{l} w=\frac{R-E\left[R\right]}{\sqrt{Var\left[R\right]}} \end{array}$$

**Figure 4:**
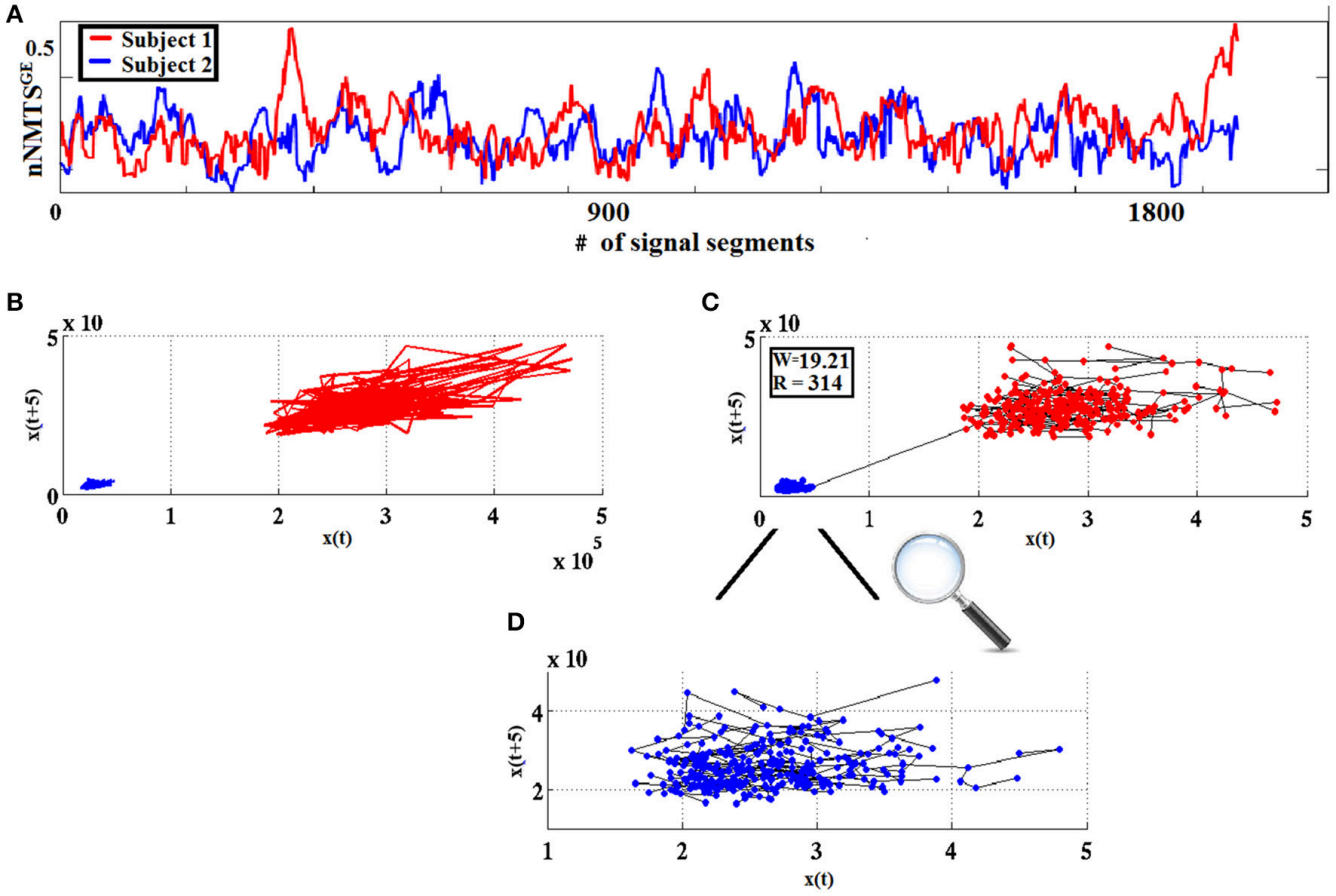
**From nNMTS^GE^ to similarity-relations: (A)** nNMTS^GE^ trace from subject 1 (blue lines) and a subject 2 (red lines); **(B)** superposition of two reconstructed trajectories (time delay = 5, embedding dimension = 3); and **(C)** using the WW test to assess similarity between the two given trajectories based on the common MST-based edges that connect the two point clouds. **(D)** Enlarged representation of the MST-based edges for the reconstructed space of nNMTS^GE^ derived from subject 1 (blue dots) shown in **(C)**.

The measure used in classification schemes in the present work was derived from w using the Heaviside step function H(x) as follows: w_dist_ = |w|.H(−w). The higher the value of w_dist_, the more dissimilar the two point-sets are considered to be. Figure 4 visualizes the WW procedure for two split half nNMTS^GE^ data obtained from two participants from frontal^θ^.

### Dynamic network connectivity analysis-based identification of individual subjects

**Figure 5:**
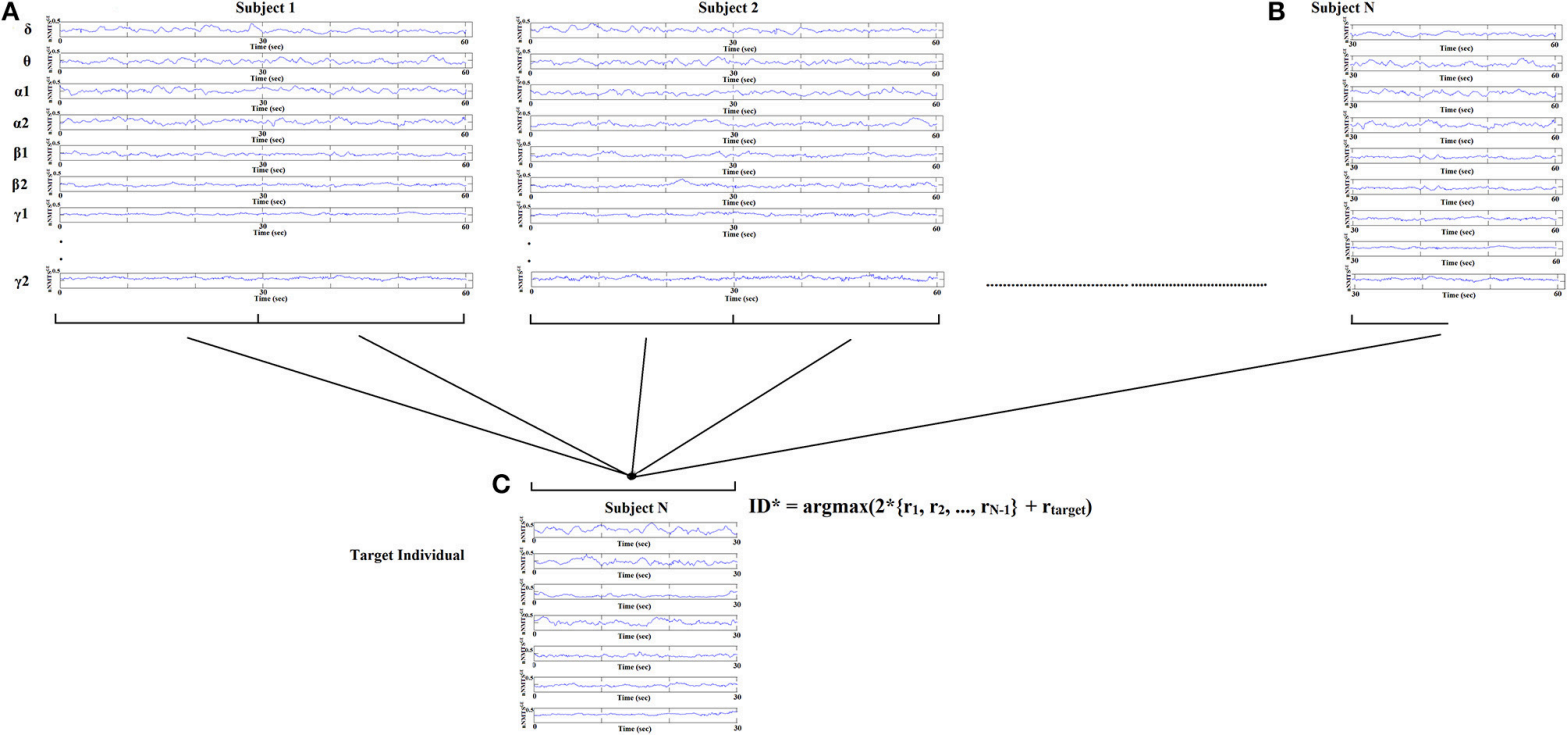
**Identification analysis procedure based on nNMTS^GE^**. Given a query of a set of individual nNMTS^GE^ from the target subject extracted from split half nNMTS^GE^, we estimated the distance between the target individual with the rest of the sets within the database. The predicted individual is the one where the majority of nNMTS^GE^ is predicted (argmax) using WW-test as a distance metric. WW-test was estimated between halves of nNMTS^GE^ from the target individual **(B)** with the two halves from the rest of the dataset **(A)** plus the second set of halves of the target individual **(C)**.

Identification of each subject was performed based on nNMTS^GE^ from EEG sensor sites and across frequency bands extracted from various thresholding schemes applied to the original DCFGs. Specifically, given a query of a nNMTS^GE^ (one of the two split nNMTS^GE^) from the target subject, we computed the correlations between this nNMTS^GE^ and all the nNMTS^GE^ in the database (2^*^100 split nNMTS^GE^ + 1 split nNMTS^GE^ from the target subject). The predicted identity ID^*^ is the one where the majority of the set of highest nNMTS^GE^ belongs to a subject (see Figure 5):

(6) $$\begin{array}{l} \text{I}{\text{D}}^{*}=\text{argmax}\left(2^{*}\left\{{\text{r}}_{1},{\text{r}}_{2},..,{\text{r}}_{\text{N}-1}\right\}+{\text{r}}_{\text{target}}\right) \end{array}$$

where r denotes the list of split nNMTS^GE^ from each subject across the sensors and frequency bands.

The objective criterion of selected the appropriate number of nNMTS^GE^ across frequency bands and sensors locations was to increase the recognition accuracy for each thresholding scheme separately.

Identification analysis procedure based on the set of selected nNMTS^GE^. Given a query of a set of individual nNMTS^GE^ from the target subject, we estimated the distance between the target individual with the rest of the sets within the database. The predicted individual ID^*^ is the one where the majority of nNMTS^GE^ belongs to using WW-test as a similarity index. WW-test was estimated between a set of halves of nNMTS^GE^ from the target individual with the two halves from the rest of the dataset. By searching all over the EEG sensors and the frequency bands, we selected the nNMTS^GE^ that improved the classification accuracy. Our features in total were 8 (frequency bands) × 64 sensors = 512 nNMTS^GE^.

To account for the contribution of various nNMTS^GE^ over prediction of individual prediction, we used a confusion matrix called CM. This CM matrix has dimensions equal to Ns × Ns where Ns denotes the number of subjects. Each row of CM matrix represents the actual class while each column represents the predicted subject S. For each individual, the identity ID was given by the Equation (6).

The recognition rate was defined as the mean of the diagonal elements of the CM matrix with the following formula:

(7) $$CM=(\frac{1}{N}\sum_{n\;=\;1}^{Ns}CM(S,S))\;\times \;100$$

## Implementation of thresholding schemes

All the threshold schemes were implemented in an in-house software toolbox using MATLAB environment and will be available from the author's website after acceptance of this manuscript. http://users.auth.gr/~stdimitr/software.html, from researchgate (https://www.researchgate.net/profile/Stavros_Dimitriadis), and from the github website (https://github.com/stdimitr/topological_filtering_networks). A webpage in scholarpedia is under construction for the promotion of the whole approach.

## Results

### EEG dataset

#### Differences of thresholding schemes in terms of shortest path lengths distribution

**Figure 6:**
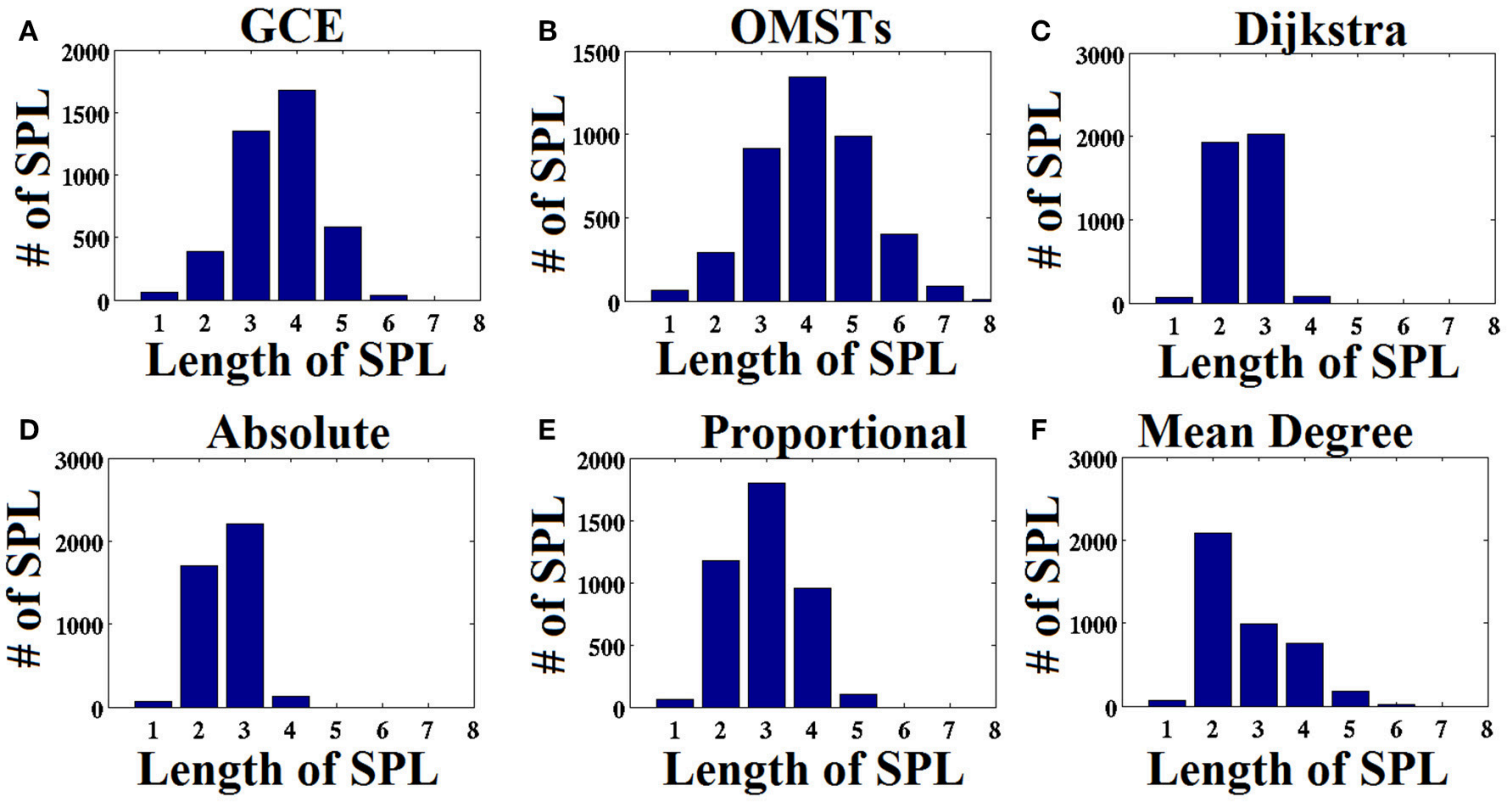
**Distribution of the length of shortest path lengths (SPL) applied to the static functional connectivity graph (FCG) thresholding by the six thresholding schemes presented in Figure 1**. **(A)** Global Cost Efficiency (GCE), **(B)** Orthogonal Minimal Spanning Trees (OMSTs), **(C)** Dijkstra's algorithm, **(D)** Absolute threshold, **(E)** Proportional Thershold, **(F)** Mean Degree Threshold. Panels **(A–C)** are data-driven topological filtering schemes while **(D–F)** are arbitrary thresholding schemes.

As an attempt to make clear the differences between the repertoire of thresholding schemes, we presented the distribution of shortest path lengths (SPLSs) after applying the six thresholding schemes to a static graph from δ band at eyes-closed condition (Figure 6). To determine the differences of the algorithms in terms of SPL, we compared the five thresholds with the one based on SPL notion (Figure 6C). The first two data-driven thresholding schemes allow more non-shortest path lengths (Figures 6A,B) compared to the arbitrary thresholds (Figures 6D–F). The explanation of this behavior is the wiring cost that is used in both data-driven methods as part of the optimized formula that allow also non-strongest connections to be part of the network compared to the arbitrary thresholding schemes where favor mostly the strongest connections.

#### Thresholding scheme alters brain fingerprinting based on nNMTS^GE^

**Figure 7:**
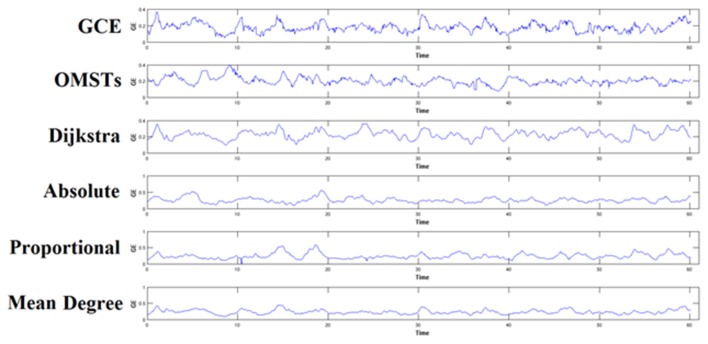
**An example of how different thresholding schemes affect the estimation of nNMTS^GE^ and finally the individual unique brain fingerprinting**. The approach was applied in δ band from Fz sensor at eyes-closed condition. GCE, Global Cost Efficiency; OMSTs, Orthogonal Minimal Spanning Trees.

To demonstrate how the adaptation of a thresholding scheme can alter the brain fingerprinting based on nNMTS^GE^, we illustrated the nNMTS^GE^ from a single-subject in δ band at eyes-closed condition from Fz sensor (Figure 7). One can clearly investigate the differences of nNMTS^GE^ across the various thresholding schemes.

#### Identification accuracy based on nNMTS^GE^

To reduce the computational effort of optimizing the threshold for the three non-data-driven thresholding schemes (absolute, proportional, mean degree), we selected a common thresholding criterion across individuals, frequencies and slides. We selected a threshold for each algorithm based on their individual criterion with main aim the highest matching between the resulted distance matrices of the thresholding graphs and the distance matrix as it was derived from the proposed algorithm of OMSTs. To estimate the distance between two graphs, Frobenius norm was used as implemented in *norm* function in Matlab. The stepping value through which the optimal matching was searched was 0.01 for absolute, 1% for proportional and 0.1 for the mean degree algorithm.

**Figure 8:**
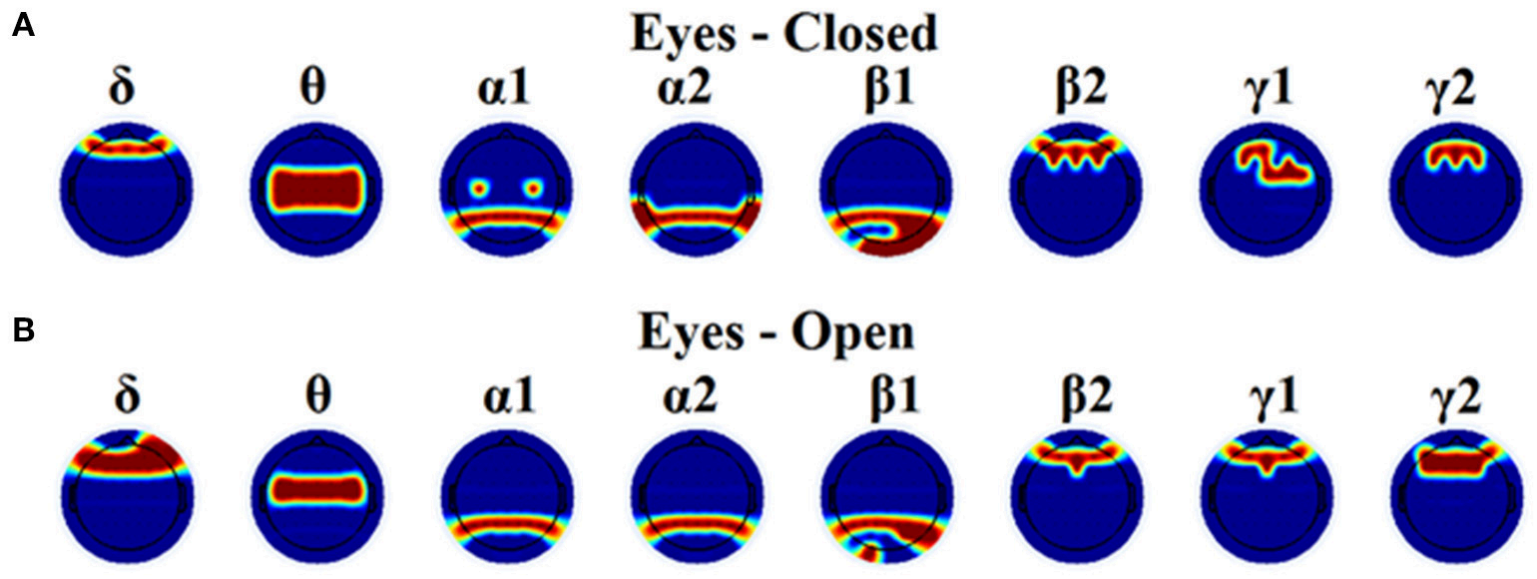
**Topographical layouts of the selected nNMTS^GE^ over frequencies for both eyes-closed (A)** and eyes-open **(B)** condition in order to get the highest accuracy for brain fingerprinting.

Table 1 tabulates the identification results for each of the thresholding schemes and also the number of selected NMTS^GE^. We also estimated the accuracy by fusing both conditions. Even though the results for such a difficult task is still high for GCE threshold, OMSTs succeeded to accurate identify all the participants with the exception of 1 on each task but with absolute accuracy by combining both tasks. Figure 8 presents the sensors across the eight frequency bands for both conditions where their nNMTS^GE^ based on OMSTs algorithm were employed as subject-specific brain fingerprinting. Tables S1–S3 (Section 2 in Supplementary Material) illustrates the identification accuracy based on the strength, the clustering coefficient and the local efficiency. Table S4 (Section 2 in Supplementary Material) demonstrates the recognition accuracy based on the global efficiency estimated on the network level, one NMTS^GE^ per frequency band.

**Table 1:** **Identification accuracy over various thresholding schemes**.

**Identification accuracy**	**GCE**	**OMSTs**	**SPL**	**Absolute**	**Proportional**	**Mean degree**
Eyes-open	0.81 (115)	0.99 (96)	0.77 (112)	0.61 (132)	0.64 (121)	0.73 (109)
Eyes-closed	0.82 (112)	0.99 (104)	0.79 (109)	0.62 (128)	0.65 (118)	0.69 (98)
Fusion	0.88	1	0.83	0.64	0.63	0.7

*In brackets, we denoted the number of the selected nNMTS^GE^ for each method and for both conditions*.

*The moving window is equal to 10cycles of the studying frequency band*.

#### The effect of window size on brain fingerprinting

It is well-known from the literature related to dynamic functional connectivity how the selection of the moving window can affect the network related metrics (Dimitriadis et al., 2010b, 2015a; Bassett et al., 2011; Hindriks et al., 2016). Here, we repeated the whole analysis by adopting 20 and 30 cycles frequency-dependent moving window and we demonstrated the result of brain fingerpring in Table 2 in association with Table 1. Clearly, we can detect the temporal stability of data-driven techniques in terms of brain fingerprinting accuracy and the number of selected features based on nNMTS^GE^ over the three widths of temporal windows.

**Table 2:** **Identification accuracy over various thresholding schemes and across two different lengths of the moving window**.

**Identification accuracy**	**GCE**	**OMSTs**	**SPL**	**Absolute**	**Proportional**	**Mean degree**
**(A) THE MOVING WINDOW IS EQUAL TO 20 CYCLES OF THE STUDYING FREQUENCY BAND**						
Eyes-open	0.84 (113)	0.99 (95)	0.75 (109)	0.57 (125)	0.61 (125)	0.68 (101)
Eyes-closed	0.85 (119)	0.99 (103)	0.75 (114)	0.58 (121)	0.62 (125)	0.65 (95)
Fusion	0.87	1	0.8	0.59	0.61	0.63
**(B) THE MOVING WINDOW IS EQUAL TO 30 CYCLES OF THE STUDYING FREQUENCY BAND**						
Eyes-open	0.81 (115)	0.99 (94)	0.74 (117)	0.57 (123)	0.61 (126)	0.66 (112)
Eyes-closed	0.82 (112)	0.99 (101)	0.75 (113)	0.56 (121)	0.6 (127)	0.64 (103)
Fusion	0.88	1	0.75	0.57	0.60	0.62

*In brackets, we denoted the number of the selected nNMTS^GE^ for each method and for both conditions*.

### fMRI dataset

**Figure 9:**
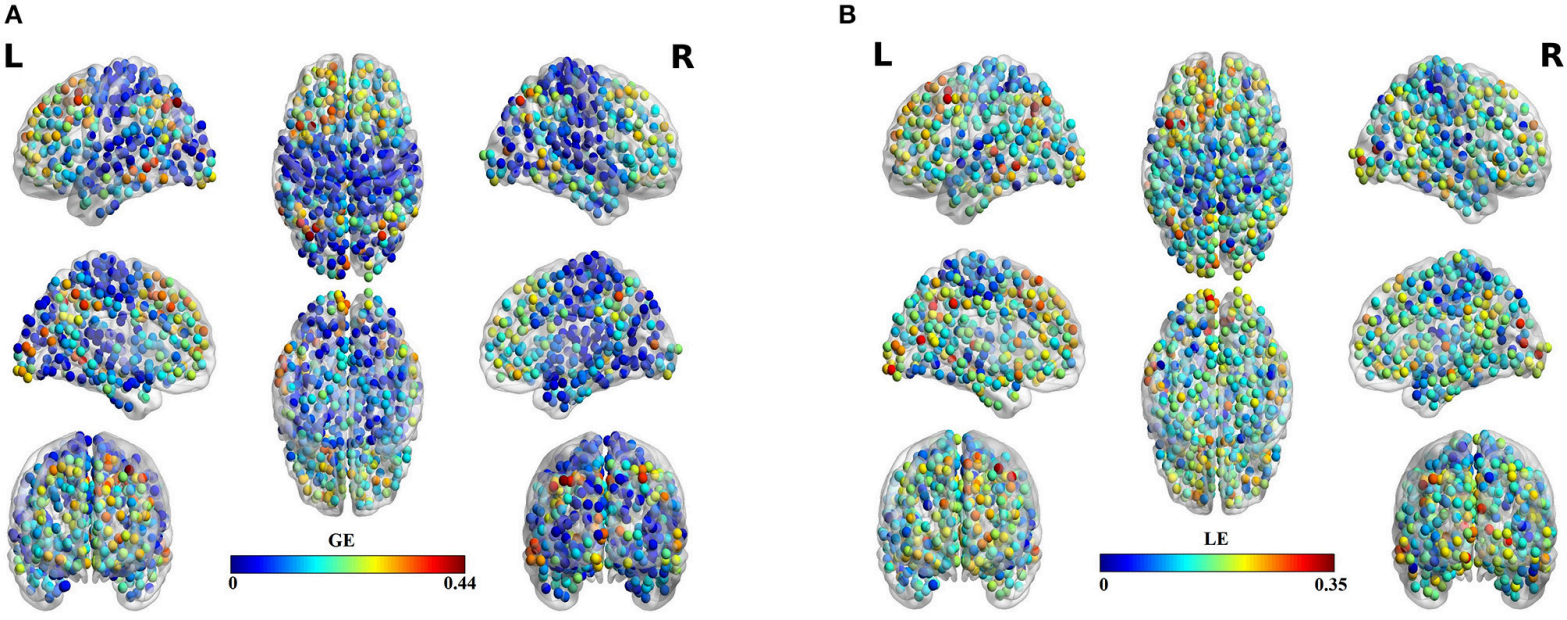
**Scan-averaged brain topographies of nodal (A)** global efficiency (GE) and **(B)** local efficiency (LE) based on the proposed data-driven topological filtering approach of OMST.

Table 3 summarizes the estimates of global and local efficiency for the six different thresholding schemes. The first three are data-driven and the rest three should be optimized under an objective criterion. Here, we used the objective criterion of increasing the ICC value. The selected absolute threshold, mean degree and % (density) are shown in brackets in Table 3. The whole analysis gave fair to good ICC scores for the 5 out of 6 thresholding schemes with the highest scores reached by the two data-driven techniques. The proposed OMST scheme demonstrated excellent reproducibility for both network metrics (ICC > 0.85). The topological layout of scan averaged nodal global and local efficiency of the fMRI dataset with the proposed OMST topological filtering scheme is illustrated in Figure 9.

**Table 3:** **Reliability of network metrics based on multiple fMRI scans**.

	**GCE**	**OMSTs**	**SPL**	**Absolute (0.235)**	**Proportional (22%)**	**Mean degree (67)**
Global Efficiency	0.34 ± 0.06 (ICC = 0.67)	0.37 ± 0.03 (**ICC** = **0.89**)	0.32 ± 0.05 (ICC = 0.71)	0.33 ± 0.11 (ICC = 0.55)	0.32 ± 0.09 (ICC = 0.56)	0.34 ± 0.08 (ICC = 0.61)
Local Efficiency	0.29 ± 0.06 (ICC = 0.71)	0.31 ± 0.03 (**ICC** = **0.91**)	0.30 ± 0.06 (ICC = 0.72)	0.31 ± 0.08 (ICC = 0.57)	0.29 ± 0.07 (ICC = 0.53)	0.31 ± 0.08 (ICC = 0.59)

*The optimized criterion for the three arbitrary thresholding schemes are presented*.

*ICC, Intra-class Correlation Coefficient*.

*We underlined in bold the highest ICC scores for each adopted network metric across the six thresholding methods*.

## Discussion

Last years, an increasing amount of human brain research based on functional imaging methods (EEG/MEG/fMRI) have adopted a more dynamic approach for exploring how brain connectivity fluctuates at resting-state and during tasks (Laufs et al., 2003; Mantini et al., 2007; Chang and Glover, 2010; Dimitriadis et al., 2010a, 2012a,c, 2013a,b, 2015a,b,c,d, 2016a,b; Bassett et al., 2011; Allen et al., 2012; Handwerker et al., 2012; Ioannides et al., 2012; Hutchison et al., 2013; Liu and Duyn, 2013; Braun et al., 2015b; Mylonas et al., 2015; Toppi et al., 2015; Yang and Lin, 2015; Calhoun and Adali, 2016, for reviews see Calhoun et al., 2014). Innovative techniques for manipulating the large number of graphs estimated via dynamic functional connectivity analysis (DFCA) have just arisen (Sakoğlu et al., 2010; Dimitriadis et al., 2013a, 2015a, 2016b; Leonardi et al., 2013; Keilholz, 2014; Leonardi and Van De Ville, 2014; Betzel et al., 2016). A crucial step in brain connectivity analysis is the application of a statistical thresholding scheme to capture the most significant connections within a graph and also the selection of a topological filtering where the most significant connections can be extracted leading to meaningful topologies. Especially, in the case of (DFCA), a data-driven approach is important in order to avoid any pitfalls in the interpretations of the results and also to enhance the reproducibility of the results of a current study under a common data-driven thresholding framework.

The majority of neuroimaging studies that followed a connectivity analysis (static or dynamic) adopted an arbitrary threshold that in many cases can vary the results in terms of group comparisons. Additionally, it restricts the usability of the results across different research groups but also within the group by adding more samples in the initial analysis. The instability of the functional network measures over different thresholds have been recently demonstrated (Garrison et al., 2015). With the increased number of open neuroimaging repositories (Sharing the wealth:Brain Imaging Repositories in 2015), it is crucial to adopt more sophisticated data-driven techniques within brain network analysis in order to aggregate the effort given by hundreds of research groups all over the world for a better understanding of how the brain functions.

In the present study, we demonstrated the superiority of a novel data-driven thresholding scheme based on OMSTs compared to the rest well-known thresholds to accurate identifies individuals in a large EEG database. The recognition accuracy was the highest in both eyes-open and eyes-closed condition compared to the rest of thresholding schemes. As unique features of this EEG biometric evaluation approach, a set of nNMTS^GE^ were used across the sensors and frequency bands. The location of the selected nNMTS^GE^ highly overlapped with the topography of the EEG spectrum related to the default mode network (DMN) of the brain (Chen et al., 2008). In δ band, there is a more prefrontal selection of the NMTS^GE^ and a more fronto-central for the θ frequency. In both α_1_ and α_2_ selected sensors were distributed at posterior parietal brain regions. In both β_1_ selected sensors were located at posterior parieto-occipital brain regions while in β_2_, γ_1_, and γ_2_ selected sensors were distributed anteriorly (Figure 8). Overall, the set of NMTS^GE^ from the whole repertoire of the eight frequency bands can be seen as the unique subject-specific brain signature of the DMN.

MST is a unique acyclic subgraph that connects all nodes with a minimal cost and also maximizes the information flow between brain regions since it captures the backbone of all shortest path lengths. One of the basic disadvantages of the MST is that it gives a sparse representation of a full-weighted functional graph. Especially, for a brain network up to or larger than 100 nodes, MST-oriented representation of the functional brain network is too sparse to get robust network metrics for characterizing the connectivity matrix. For that reason, the method of OMSTs with the objective criterion of maximizing the information flow with the restriction of the wiring cost presents a more robust approach. Here, we demonstrated the superiority of OMSTs over the existing methods of an EEG dynamic functional connectivity analysis at resting-state with the main scope to maximizing the identification accuracy within a database.

The basic explanation of the superiority of the proposed algorithm over the rest and especially the data-driven techniques (Bassett and Bullmore, 2006) is the sampling of weighted connections with main constrain to connect all the nodes with minimal cost without ranking connections according to their strength. The algorithm presented by Bassett et al., searches over the network by windowing the range of the weights which are often between 0 and 1 starting from 0 up to 1 with a small stepping criterion (e.g., 0.01; Bassett and Bullmore, 2006). This option separates strongest from weakest connections obliviously also if the network is connected. It seems that MST and especially multiple rounds of OMSTs is a reliable sampling approach for the selection of the subgraph that better described the functionality of the studying brain topology. A recent study combined MST with an arbitrary proportional threshold to get the sparse representations of brain networks (Song et al., 2015).

One significant observation of our method is that using the penalty term of wiring cost, part of information flows via non-shortest path lengths compared to the notion of shortest path lengths (see Figure 6; Van Mieghem and Wang, 2009; Dimitriadis et al., 2010a, 2012b). Our previous data-driven algorithm based on shortest-path lengths have already been reported as the Union of Shortest Path Trees (USPL; Van Mieghem and Wang, 2009) and applied to functional brain networks (Meier et al., 2015).

It is important to mention here that our main goal was to present the superiority of our method overall the existing thresholding schemes in a large EEG database by adopting a dynamic functional connectivity analysis. The objective criterion to validate the proposed data-driven thresholding scheme based on OMSTs was the identification accuracy of each individual further supporting recent efforts over functional connectome brain fingerprinting (Finn et al., 2015). Additionally, previous attempts have already demonstrated high classification accuracy based on power spectrum and coherence on static graphs on the same database (La Rocca et al., 2014). Our approach improves current efforts over EEG-based biometric systems by extracting meaningful temporal graph-oriented features supporting and validating the proposed data-driven threshold algorithm (OMSTs) via the notion of brain fingerprinting. Finally, we demonstrated its temporal stability in terms of recognition accuracy and the number of selected features over three different widths of temporal windows.

Finally, the proposed data-driven thresholding scheme based on OMSTs demonstrates the need of a data-driven approach for topological filtering of brain networks. Complementary, it shows that sampling the connections in weighted brain network using orthogonal MSTs is a superior approach compared to taking into consideration only their strength (Bassett and Bullmore, 2006). Additionally, the wiring cost is a significant attribute of a brain network while it was proved its key role to the evolution of hierarchy and modularity by its direct link to sparsity (Mengistu et al., 2016). Further studies should validate the superiority of this approach to multimodal imaging and to the design of reliable connectomic biomarkers for various brain disorders/diseases.

The analysis of functional brain networks derived from resting-state recordings or task-related via graph theory has been proven to be a powerful tool to characterize both globally and locally the architecture of functional connectivity in the human brain (Van Dijk et al., 2010). Due to the increment of research studies that expanded their results based on network analysis to daily clinical use (Savitz et al., 2013), it is significant to provide data-driven solutions of preprocessing steps in order to increase the reproducibility of the network metrics (Zuo and Xing, 2014; Zuo et al., 2014; Chen et al., 2015). Here, we investigated how the different topological filtering scheme (data-driven vs. arbitrary algorithms) can alter the reliability of basic network metrics estimated via ICC score. Apart from the choice of topological filtering scheme of functional brain network, the selection of the connectivity estimator can also affect the reliability of the network metrics (Garcés et al., 2016). The effect of both the connectivity estimator and the selection of filtering schemes has been recently demonstrated (Jalili, 2016) and we will present the reproducibility of network metrics based on brain connectivity on the source level with both EEG and MEG recordings in a next upcoming journal paper.

Employing a second fMRI case study dataset and following a static network analysis, we demonstrated the superiority of the proposed topological filtering scheme to increase the test-retest reliability of the network metrics across different scans/sessions (Deuker et al., 2009). The reliability of network metrics across trials is an important issue in order to make general conclusions for a targeted group (e.g., Alzheimer) or a task (e.g., resting-state, n-back memory etc.).

## Limitations and further validations

To further validate the proposed thresholding scheme over the highly used thresholds, it is important to apply it also on EEG and MEG source level (Ioannides et al., 2012) and also to fMRI experimental paradigms where the spatial resolution is higher compared to the scalp-recorded EEG activity. Additionally, it is significant to compare nNMTS between resting-state and tasks in both control and disease groups (Damaraju et al., 2014) but also during the developmental time (Dimitriadis et al., 2015a). Finally, by adopting multivariate estimators like Granger causality or partial direct coherence (Omidvarnia et al., 2013) in a dynamic fashion with the proposed algorithm may further shed light on the importance of data-driven approaches in neuroimaging.

It will be interesting in the future to generate brain network models of the human connectome (Betzel et al., 2016) based on the notion of OMSTs using a distance geometric penalty (e.g., Euclidean distance between ROIs in 3-dimensional space). It seems that geometry is crucial in the studying of structural connectome (Roberts et al., 2016), an observation that should be further validated if it contributes to the network topology in functional connectome at source space.

## Concluding remarks

We showed that a data-driven thresholding scheme based on OMSTs implies a unique description of each individual brain activity based on the notion of nodal network metrics times series and namely the global efficiency (nNMTS^GE^). The proposed technique overcame the rest of highly used thresholding schemes in terms of human brain distinctiveness employing a dynamic functional connectivity approach. Moreover, we demonstrated its effectiveness to increase the reliability of network metrics in a multi-scan fMRI dataset. The novel method has practical significance over the existing thresholding schemes, in that it represents a model-free framework for identifying the significant connections over which the information flow between the brain areas is maximized with the constraints of the wiring cost. Current approach could be of greater neuroinformatic importance especially in the direction of reproducibility of brain connectivity results across different data repositories based on various imaging methods like EEG,MEG and fMRI. Especially, in a simultaneous recording of different imaging methods (MEG-EEG and EEG-fMRI) the proposed algorithm could be a common framework to compare dynamic FCGs estimated from two different modalities. The proposed method deals with a data-driven filtering of (chro)/(co)nnectomics.

## Author contributions

Conception of the research: SD; Methods and design: SD; Data analysis (SD, CS); Drafting the manuscript: SD; Critical revision of the manuscript: IT, DL; Every author read and approved the final version of the manuscript.

## Funding

This study was supported by the National Centre for Mental Health (NCMH) at Cardiff University. We would like to acknowledge Cardiff RCUK funding scheme.

### Conflict of interest statement

The authors declare that the research was conducted in the absence of any commercial or financial relationships that could be construed as a potential conflict of interest.

## Footnotes

^1^https://physionet.org/pn4/eegmmidb/

^2^https://github.com/poldrack?tab=repositories
