# Supplementary material for: Topological Filtering of Dynamic Functional Brain Networks Unfolds Informative Chronnectomics: A Novel Data-Driven Thresholding Scheme Based on Orthogonal Minimal Spanning Trees (OMSTs)
Source: Front Neuroinform. 2017 Apr 26;11:28. doi: 10.3389/fninf.2017.00028 (PMC5405139; doi:10.3389/fninf.2017.00028)
Supplement: Supplementary file 1 [file DataSheet1.docx]

Topological Filtering of Dynamic Functional Brain Networks Unfold Informative Chronnectomics: A novel data-driven thresholding scheme based on Orthogonal Minimal Spanning Trees (OMSTs)

S. I. Dimitriadis^1,2,3,4*^, Salis C^5^, I.Tarnanas^6,7^,D.Linden^1,2,8^

^1^ Institute of Psychological Medicine and Clinical Neurosciences, Cardiff University School of Medicine, Cardiff, United Kingdom

^2^ Cardiff University Brain Research Imaging Center (CUBRIC), School of Psychology, Cardiff University, Cardiff, United Kingdom

^3^ School of Psychology, Cardiff University, Cardiff, CF24 4HQ ,United Kingdom

^4^ Neuroinformatics.Group, School of Psychology, Cardiff University, Cardiff, CF24 4HQ,United Kingdom

^5^ University of Western Macedonia, Department of Informatics and Telecommunications Engineering

^6^Health-IS Lab, Chair of Information Management, ETH Zurich, Zurich, Switzerland

^7^3rd Department of Neurology, Medical School, Aristotle University of Thessaloniki, Thessaloniki, Greece

^8^ Neuroscience and Mental Health Research Institute (NMHRI), School of Medicine, Cardiff University, Cardiff, United Kingdom

***Corresponding author:**

Dr.Dimitriadis Stavros

Research Fellow with

Institute of Psychological Medicine and Clinical Neurosciences, Cardiff University School of Medicine, Cardiff, United Kingdom

Cardiff University Brain Research Imaging Center (CUBRIC), School of Psychology, Cardiff University, Cardiff, United Kingdom

Tel:+44 – 0785 198 3732

Fax: +44 (0)29 208 70339

Email:DImitriadisS@cardiff.ac.uk; stidimitriadis@gmail.com

Cardiff University Brain Research Imaging Centre
School of Psychology
Cardiff University
Maindy Road
Cardiff
CF24 4HQ

**1.Delay Vector Variance and Surrogates**

The Delay vector variance (DVV) method uses predictability of the signal in phase space to characterize the time series. Using the surrogate data methodology, so called DVV plots and DVV scatter diagrams can be generated using the DVV method, as a test statistic, to examine the determinism/stochastisity and linearity/nonlinearity within a signal simultaneously. In DVV scatter diagram, the target variance values of the original signal is plotted against the averaged variance values, calculated over a number of iAAFT surrogates (Gautama et al., 2004). As a result, for linear signals, the scatter diagram coincides with the bisector line and conversely for nonlinear signals, the scatter diagram deviates from bisector line as shown in the S1.

In S1, we demonstrated how the method of creation of surrogates can affect their determinism and nonlinearity. S1.a demonstrates the degree of nonlinearity of the original time series deviated from the surrogates used in DVV method. S1.b shows the preservation nonlinearity in the surrogates created by cutting at a single point at a random location the original time series and exchanging the two resulting time courses (Aru et al., 2014). Contrary, the nonlinearity destroyed in surrogates created by shuffling the original time series. It is important to mention here that surrogates used in the DVV method are different compared to the surrogates used for creation a baseline for connectivity analysis.


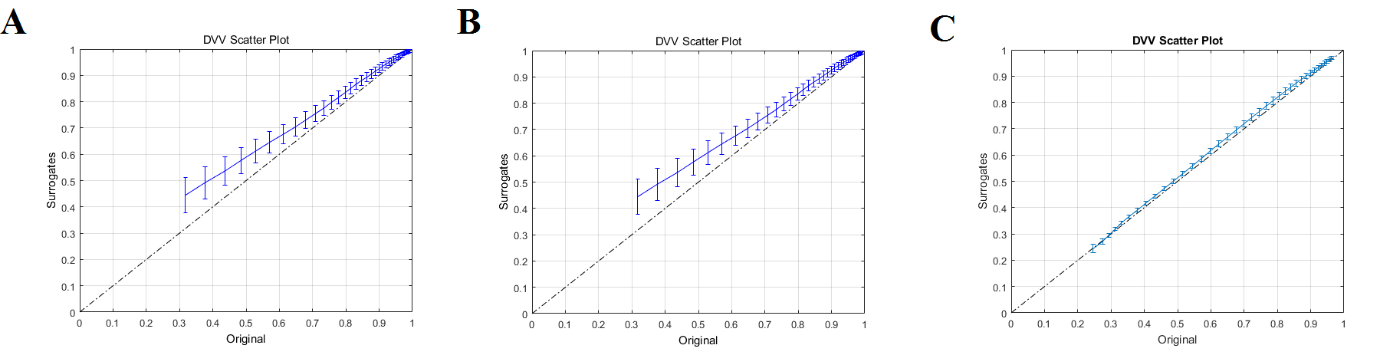


**S1. Estimated nonlinearity with DVV of original time series and the related surrogates using two approaches.**

1. Nonlinearity of original time series
2. Nonlinearity of surrogates using a random point
3. Nonlinearity of surrogates created by shuffling the original time series

**2.Identification Accuracy Using Alternative Network Metrics and Global Network Metric Time Series**

Apart from network metric time series (NMTS) based on nodal global efficiency (nGE), we employed alternative nodal network metrics such as the strength of each node (total sum of iPLV weights of survived functional connections between each node and the rest of the network), the clustering coefficient and the local efficiency. In all three cases, the highest identification accuracies were succeeded by the data-driven topological filtering schemes and especially with the proposed ones (see S.Table 1-3). Complementary, the highest performance was attained by local efficiency.

We also used one global NMTS per frequency band based on the estimation of GE for the whole network. Stable 4 tabulates the results of the recognition accuracy across the thresholding schemes based on the 8 NMTS^GE^. The results revealed a low identification accuracy for the GE in the network level with highest performance ( < 50 %) for the OMST. This result further enhanced the need of studying networks on a node level.

**Stable 1.** Identification Accuracy over various thresholding schemes for the strength (STR).

In brackets, we denoted the number of the selected NMTS^STR^ for each method and for both conditions.

The moving window is equal to thirty cycles of the studying frequency band.

| **Identification Accuracy** | **GCE** | **OMSTs** | **SPL** | **Absolute** | **Proportional** | **Mean Degree** |
| --- | --- | --- | --- | --- | --- | --- |
| **Eyes-open** | 0.59 (102) | 0.68 (99) | 0.54 (105) | 0.40 (87) | 0.43 (107) | 0.45 (101) |
| **Eyes-closed** | 0.55 (96) | 0.69 (88) | 0.52 (103) | 0.41 (91) | 0.47 (103) | 0.43 (98) |
| **Fusion** | 0.58 | 0.70 | 0.55 | 0.42 | 0.48 | 0.46 |

**Stable 2.** Identification Accuracy over various thresholding schemes for the clustering coefficient (CC).

In brackets, we denoted the number of the selected NMTS^CC^ for each method and for both conditions.

The moving window is equal to thirty cycles of the studying frequency band.

| **Identification Accuracy** | **GCE** | **OMSTs** | **SPL** | **Absolute** | **Proportional** | **Mean Degree** |
| --- | --- | --- | --- | --- | --- | --- |
| **Eyes-open** | 0.53 (78) | 0.65 (74) | 0.51 (95) | 0.38 (85) | 0.42 (101) | 0.47 (87) |
| **Eyes-closed** | 0.51 (81) | 0.63 (81) | 0.48 (88) | 0.37 (88) | 0.44 (95) | 0.42 (91) |
| **Fusion** | 0.54 | 0.66 | 0.50 | 0.39 | 0.43 | 0.45 |

**Stable 3.** Identification Accuracy over various thresholding schemes for the local efficiency (LE).

In brackets, we denoted the number of the selected NMTS^LE^ for each method and for both conditions.

The moving window is equal to thirty cycles of the studying frequency band.

| **Identification Accuracy** | **GCE** | **OMSTs** | **SPL** | **Absolute** | **Proportional** | **Mean Degree** |
| --- | --- | --- | --- | --- | --- | --- |
| **Eyes-open** | 0.64 (89) | 0.81 (92) | 0.58 (98) | 0.53 (103) | 0.50 (112) | 0.54 (92) |
| **Eyes-closed** | 0.65 (87) | 0.80 (105) | 0.57 (103) | 0.51 (101) | 0.52 (118) | 0.53 (89) |
| **Fusion** | 0.67 | 0.78 | 0.57 | 0.52 | 0.52 | 0.54 |

**Stable 4.** Identification Accuracy over various thresholding schemes for the glocal efficiency (GE) in the network level.

In brackets, we denoted the number of the selected NMTS^GE^ (from the 8 ; one for each frequency band) for each method and for both conditions.

The moving window is equal to thirty cycles of the studying frequency band.

| **Identification Accuracy** | **GCE** | **OMSTs** | **SPL** | **Absolute** | **Proportional** | **Mean Degree** |
| --- | --- | --- | --- | --- | --- | --- |
| **Eyes-open** | 0.42 (8) | 0.49 (8) | 0.35 (8) | 0.30 (8) | 0.31 (8) | 0.28 (8) |
| **Eyes-closed** | 0.40 (8) | 0.48 (8) | 0.36 (8) | 0.34 (8) | 0.32 (8) | 0.27 (8) |
| **Fusion** | 0.41 | 0.47 | 0.36 | 0.32 | 0.33 | 0.29 |

**References**

Aru J, J. Aru, V. Priesemann, M. Wibral, L. Lana, G. Pipa, *et al.*Untangling cross-frequency coupling in neuroscience**.** Curr Opin Neurobiol, 31C (2014), pp. 51–61

Gautama,T D.P. Mandic, and M. M. Van Hulle. **The delay vector variance method for detecting determinism and nonlinearity in time series.** Physica D, vol. 190, no. 3-4, pp. 167-176, 2004.
